# Generalisation of EEG-Based Pain Biomarker Classification for Predicting Central Neuropathic Pain in Subacute Spinal Cord Injury

**DOI:** 10.3390/biomedicines13010213

**Published:** 2025-01-16

**Authors:** Keri Anderson, Sebastian Stein, Ho Suen, Mariel Purcell, Maurizio Belci, Euan McCaughey, Ronali McLean, Aye Khine, Aleksandra Vuckovic

**Affiliations:** 1 Biomedical Engineering Division, James Watt School of Engineering, University of Glasgow, Glasgow G12 8QQ, UK; 2 School of Computing Science, University of Glasgow, Glasgow G12 8QQ, UK; sebastian.stein@glasgow.ac.uk; 3 Queen Elizabeth National Spinal Injuries Unit, Queen Elizabeth University Hospital, Glasgow G51 4TF, UK; 4 Stoke Mandeville Spinal Injuries Centre, Stoke Mandeville Hospital, Aylesbury HP21 8AL, UKaye.khine@nhs.net (A.K.)

**Keywords:** EEG, central neuropathic pain, spinal cord injury, biomarkers, machine learning

## Abstract

**Background:** The objective was to test the generalisability of electroencephalography (EEG) markers of future pain using two independent datasets. **Methods:** Datasets, A [N = 20] and B [N = 35], were collected from participants with subacute spinal cord injury who did not have neuropathic pain at the time of recording. In both datasets, some participants developed pain within six months, (PDP) will others did not (PNP). EEG features were extracted based on either band power or Higuchi fractal dimension (HFD). Three levels of generalisability were tested: (1) classification PDP vs. PNP in datasets A and B separately; (2) classification between groups in datasets A and B together; and (3) classification where one dataset (A or B) was used for training and testing, and the other for validation. A novel normalisation method was applied to HFD features. **Results:** Training and testing of individual datasets achieved classification accuracies of >80% using either feature set, and classification of joint datasets (A and B) achieved a maximum accuracy of 86.4% (HFD, support vector machine (SVM)). With normalisation and feature reduction (principal components), the validation accuracy was 66.6%. **Conclusions:** An SVM classifier with HFD features showed the best robustness, and normalisation improved the accuracy of predicting future neuropathic pain well above the chance level.

## 1. Introduction

Central neuropathic pain (CNP) is a debilitating condition that frequently follows spinal cord injury (SCI), significantly decreasing patients’ quality of life [1,2,3]. Electroencephalography (EEG) has large potential for identifying diagnostic and susceptibility (future pain) [4] biomarkers for CNP given its cost-effective, portable, and non-invasive nature. Various models have shown great promise in explaining pain-predictive models [5,6], though there remains a pressing need to improve biomarkers and their classification accuracy. A study by our group demonstrated that SCI-related CNP could be predicted using oscillatory EEG features with accuracies exceeding 80% [7]. Studies such as these, based on small datasets, provide early evidence for potentially clinically useful biomarkers but lack validation on independent datasets, making it unclear whether models would hold on similar datasets and leading to uncertainty regarding the generalisability of findings.

Various studies have utilised spontaneous cortical activity, using resting state EEG spectral analysis, to compare between patients with pain relating to neurological diseases and patients with the same disease but without pain or even healthy controls [8,9,10,11,12,13,14,15,16]. A review of resting-state biomarkers of chronic NP revealed that chronic neuropathic pain was associated with EEG signal power increases in the theta and high-beta bands, but a decrease in the high alpha–low beta band [17]. More specifically to SCI-related CNP, studies have observed similar changes in the oscillatory activity of the brain during spontaneous EEG that relate to pain [18,19,20,21,22]. The main findings of these studies indicate that major signatures of CNP include increased power in the theta range and a decreased frequency of the dominant alpha rhythm. These observed changes in EEG power are widespread and not restricted to specific cortical areas. It is these markers that have led to the conclusion that CNP may be related to thalamocortical dysrhythmia (TCD), in which normal resonance is disrupted by changes in the behaviour of the neurons in the thalamus [18]. This dysrhythmia in CNP is characterised as an increase in theta and higher beta band EEG power, as well as shifted dominant alpha frequencies towards lower values [8,21].

Non-linear methods, originating from chaos theory (e.g., fractal dimension), provide unique insights into EEG signals by measuring signal complexity [23,24,25]. Higuchi’s fractal dimension (HFD) has been widely applied to EEG studies in both healthy individuals and those with neurological conditions, including Alzheimer’s, traumatic brain injury, and epilepsy [25,26,27]. There is little evidence of studies implementing HFD to analyse cortical changes due to pain; however, similar studies have used resting-state EEGs to confirm decreased HFD in Alzheimer’s disease as a classifiable biomarker, with the results of one support vector machine (SVM) classifier achieving sensitivity and specificity above 90% [28]. Notably, a recent study carried out by our group investigated the development of CNP in a subacute SCI population, applying HFD to dynamic EEG signals and classifying between those who did and did not develop CNP using SVM models and obtaining accuracies of up to 88% [29].

In recent years, there has been lots of interest in applying machine learning methods to analyse changes in brain activity, using EEG, in order to both identify pain and predict future pain [30,31,32,33,34].

Chronic pain has been classified using EEG-based biomarkers for distinguishing between people with and without hip pain [35], chronic pancreatitis [36], lower back pain [15,32], peripheral neuropathy [37], herpes zoster neuropathy [37], chronic pain following traumatic brain injury [38], phantom limb pain [39], migraine [40], or mixed types of pain [41,42,43]. Classification accuracies ranged from 57% [41] to 92.5% [42].

While SVM has been the most popular classifier, several studies have used neural networks (NNs) such as perceptron NNs for detecting neuropathic pain [7], convolutional NNs for induced back pain [32], elastic net for mixed chronic pain [43], and STPA Net for detecting the presence of pain in children [44]. For NNs, classifiers’ accuracies ranged from 79.6 [43] to 89% [7]. Classifiers based on NNs did not provide noticeably higher classification accuracies than simple machine learning methods, probably because all datasets were relatively small. While NNs might provide flexibility in the sense of feature selection, they have a large number of hyperparameters and might be prone to overfitting.

What is common for all studies is that they typically focus on diagnostic markers, i.e., confirming the presence of existing pain, which may reveal new pain markers and contribute to our understanding of pain mechanisms, although this is not of great relevance for patients. Secondly, studies typically use cross-validation to test accuracy, i.e., to test the robustness of the classifiers.

It has been suggested that standard criteria should be identified and met to determine markers as valid and useful for clinical practice [45]. Good biomarkers need to be generalisable and interpretable, transparently follow clear standardised procedures, and have high sensitivity and specificity [6]. The generalisability of biomarkers refers to whether a prediction will hold when applied to different test subjects or datasets. One survey of translational neuroimaging studies, not limited to EEG studies, found that only a small portion (9%) of over 500 studies used prospective testing on new datasets to validate developed classification models for various neurological disorders [46]. Studies that did conduct independent testing often reported lower accuracies, potentially due to biased cross-validation estimates derived from original training data where small datasets are often described as major limitations in neuroimaging-based classification studies.

There are two novelties of this study. First, it focuses on identifying people who in the future might develop pain and second, it validates classifiers on unseen data. Rather than using cross-validation, we explore both linear and non-linear EEG features as candidate susceptibility markers, i.e., markers of the risk for developing neuropathic pain in the future.

We hypothesise that future pain can be identified by both linear and non-linear EEG markers and that these markers are robust when testing on unseen datasets. We test this hypothesis on patients with subacute spinal cord injury (SCI, within 6 months post-injury), recording their EEG prior to developing NP, and following up their pain status. The specific group of patients was selected because 50% of people with SCI develop pain within the first 6 months [1]. Although by definition, chronic pain should be present for at least 6 months, the specific location (under the level of injury) and responses to sensory tests allow for CNP in SCI to be diagnosed earlier [47].

The specific objectives of the study are as follows:

- Compare the classification performance of linear (band power) and non-linear (HDF) EEG features as markers of pain using two frequently used classifiers (LDA and SVM) as potential makers of future NP.
- Explore the robustness of features and classifiers by gradually increasing the level of challenge: (1) when applied for training and testing on two different datasets (A and B separately); (2) training and testing on the combined dataset (A and B); and (3) training and testing on one dataset (A or B) and validating on another dataset (B or A) separately.

## 2. Materials and Methods

### 2.1. Datasets

Practical purpose classifiers should be able to classify datasets provided in different laboratories or clinics with high accuracy. We compared classification accuracy on two independent datasets, which were recorded 4 years apart by different experimenters under the same experimental protocol and using the same EEG device in different environments. This mimicked to some extent the realistic conditions limiting, in the first instance, variability of the external factors.

#### 2.1.1. Dataset A

A pre-existing dataset of EEG recordings was used in this study. This study was part of a registered clinical trial (NCT021789917) [7]. The dataset includes paraplegic and tetraplegic subacute (≤6 months post-injury) spinal cord-injured patients. Data were recorded with a 48-channel EEG at 256 Hz and involve three groups of participants:

- Ten able-bodied (AB) participants (three female (F), seven male (M), age 35.2 ± 7.2 years [mean ± std.dev])
- Ten patients who eventually developed pain (PDP) within six months of EEG recording (one F, nine M, age 46.9 ± 15.9 years).
- Ten patients who did not develop pain (PNP) within six months of EEG recording (one F, nine M, age 42.1 ± 13.3 years)
- Eleven patients with pain (PWP) at the time of EEG recording (four F, seven M, age 44.9 ± 16.9 years)

Participants were considered to have pain if their pain level was ≥4 on the visual numerical scale (where zero is no pain and ten corresponds to the worst pain imaginable). The location of EEG electrodes in both datasets is shown in the Supplementary Materials (Figure S1). Note that, in Dataset B, only electrodes present in Dataset A were used.

#### 2.1.2. Dataset B

A dataset consisting of 75 participants with SCI and AB controls was collected, annotated, and preprocessed for this study via a multi-site clinical trial (registered clinical trial number NCT04665492). Researchers performed data collection using the same portable EEG device at both sites, and the same device was used for recording Dataset A (g.USBamp; Guger Technologies, Graz, Austria). Half of the participants’ data in Dataset B was recorded in the same environment as Dataset A. The inclusion/exclusion criteria were the same as for Dataset A. General inclusion criteria for all participants were as follows: age between 18 and 75 years old, no known other major neurological disorder or injury that would affect EEG interpretation and the ability to understand the task. The general exclusion criteria for all groups was the presence of any chronic (non-CNP) or acute pain at the time of EEG recording. All SCI patients in this study were within 6 months of injury, still hospitalised, and receiving inpatient rehabilitation for their SCI. Given that there is no known or confirmed relation between development of NP and age, gender, and level or completeness of injury [48], participants with SCI of any sex and incomplete injury level or severity were included in the study, similarly to the recruitment criteria in the preceding study that collected Dataset A [49]. There were two groups of patients: those who already had below-level neuropathic pain at the time of recording and patients who did not have neuropathic or any other chronic pain at the time of the EEG recording. Patients who did not have any pain at recording were followed up after approximately six months to identify whether they had been diagnosed with CNP. Pain status was based on patient records and phone calls by medical professionals. In both datasets, the presence of NP was established based on (1) a complete patient history, (2) a physical examination, and (3) the International Spinal Cord Injury Pain (ISCIP) Classification System following best practice for establishing the presence of NP in subacute SCI [47]. After this period, they were further divided into a group who eventually developed neuropathic pain and a group who did not develop pain. Participants were therefore divided into the same four groups as in Dataset A for EEG analysis:

1. Twenty AB participants (five F, fifteen M, age 51.2 ± 12.8 years)
2. Seventeen PDP participants(five F, twelve M, age 56.9 ± 12.4 years)
3. Fifteen PNP participants (one F, fourteen M, age 57.5 ± 18.2 years)
4. Nineteen PWP participants (eight F, eleven M, age 45.9 ± 15.6 years)

Though the same methods could be applied to all combinations of groups, this study only analyses the PDP and PNP groups where successfully distinguishing between them, early after injury, would be of clinical significance in identifying patient susceptibility to pain.

Information about patients in Datasets A and B is shown in the Supplementary Materials in Table S1. In both groups A and B, PDP had more patients with completeness of injury A and B and PNP had more patients with completeness of injury C and D. Note that this was not known at the time of EEG recording, because patients were split post-hoc into PNP and PDP. The flow chart in Supplementary Materials Figure S2 shows all patient groups in Datasets A and B and the patient groups analysed in this study.

The experimental protocol (Nr GN20NE240) was approved by the Research Ethics Committee (REC) of the National Health Services (NHS) for Scotland. Each participant signed the informed consent form, and the study was carried out in accordance with the Declaration of Helsinki (1964). For more details about the studies, please look at publicly available registered trials.

### 2.2. EEG Recordings

During the recording of both datasets, the electrode impedance was kept under 5 kΩ. The ground electrode was placed at the AFz electrode location in Dataset A and at FPz in Dataset B. Both datasets were recorded using a linked-ear reference. EEG was sampled at 256 Hz, band-pass filtered during recording between 0.5–60 Hz and notch filtered at 50 Hz, using fifth order IIR digital Butterworth filters within the g.USBamp devices.

All recordings were imported to Matlab for preprocessing using the EEGLAB toolbox [50]. Data were high-pass filtered at 1 Hz using bandpass FIR filter, which was necessary in order to implement ICA. The EEG signal was re-referenced to an average reference. Next, signals were visually inspected and segments with artifacts of an amplitude ≥100 μV across all electrodes were manually removed. For further artifact removal, the remaining data were decomposed into independent temporal components (corresponding to the number of available EEG channels) using the Infomax ICA algorithm [51], implemented in EEGLAB. The non-EEG components were identified visually and removed by considering their frequency content, morphology, and spatial distribution [52].

### 2.3. Experimental Paradigm

Spontaneous EEG activity was recorded in the eyes-opened (EO) and eyes-closed (EC) relaxed states for two minutes each. During the EO relaxed state, participants were presented with a small cross on a computer screen to focus on and were instructed to stay as still as possible during recording. Similarly, during the EC relaxed state, participants were asked to relax. A 100 s long EEG recording was taken from the EO and EC data of each participant, based on the shortest available EEG across both datasets for analysis.

### 2.4. Feature Extraction

Oscillatory and non-oscillatory features were extracted and used independently for classification in this study, where analyses based on both have previously shown promising classification accuracies between participant groups in Dataset A [7,29].

#### 2.4.1. Band Power

For both datasets, EEG data were divided into 10 equal, non-overlapping, subsequent sequences, each 10.0 s long (100.0 s in total), from which features were extracted from each sequence independently. For every participant (N) in each group, this created 10*N training sets for the EO and EC states seperately. All extracted features were based on the power of the EEG signals in various frequency bands. The power spectrum density was calculated based on Welch’s periodogram over 4 s windows with 50% overlap.

The *compute_psd()* function from MNE-Python (mne-1.8.0) was utilised to estimate the power spectral density (PSD) using Welch’s method. The frequency range was specified between 2 Hz and 30 Hz. Both absolute band power and relative band power were derived for each frequency band of interest in order to evaluate the PSD. The absolute power for a given frequency band was calculated as illustrated in Equation (1):

(1) $$\text{absolute\_bandpower}=\int_{f_{\mathrm{low}}}^{f_{\mathrm{high}}}\mathrm{psd}\left(f\right)\;df$$

Here, $f_{\mathrm{low}}$ and $f_{\mathrm{high}}$ represent the low and high cut-off frequencies, respectively. To calculate the relative band power, the absolute band power was normalised by dividing it by the total power across all frequencies. The total power is obtained by integrating the full PSD spectrum, as shown in Equation (2):

(2) total_power=∫psd(f)df

The relative band power can then be expressed as:

(3) $$\text{relative\_bandpower}=\frac{\mathrm{bandpower}}{\text{total\_power}}$$

By normalising against the total power, the relative power highlights the proportion of power contained within a specific frequency band relative to the overall EEG spectrum.

EEG power for both EO and EC states was calculated in the theta (4–8 Hz), alpha (8–12 Hz), beta (13–30 Hz), and wide band (2–30 Hz) ranges. Relative power was calculated, for normalisation purposes, by dividing the power in each frequency band (theta, alpha, beta) by the wide band power. The resulting feature vector inputs for classification are therefore:

- EO theta, alpha, beta
- EC theta, alpha, beta

#### 2.4.2. Higuchi Fractal Dimension

Higuchi’s fractal dimension (HFD) is also explored as a classifiable feature to understand whether non-linear analysis provides discriminatory information between the two groups [25]. The fractal dimension *D* is a statistical measure relating signal complexity to the scale at which a signal is measured, with higher values of *D* corresponding to higher signal complexity. For time series data, D∈[1,2].

The HFD is estimated as follows. First, the length L(k) of a time series S(t), t=1,…,N is calculated using Equations (4) and (5) for exponentially increasing values of k=2,…,≤$k_{max}$.

(4) $$\begin{array}{cc} L_{m}\left(k\right) & =\left(\sum_{i=1}^{\left\lfloor \frac{N-m}{k}\right\rfloor }\left|S\left(m+ik\right)-S\left(m+\left(i-1\right)k\right)\right|\right)\frac{1}{k}\frac{N-1}{\left\lfloor \frac{N-m}{k}\right\rfloor } \end{array}$$

(5) $$\begin{array}{cc} L(k) & =\frac{1}{k}\sum_{m=0}^{k-1}L_{m}\left(k\right) \end{array}$$

Then, the fractal dimension *D* is estimated from the slope of the linear least squares fit of L(k) onto *k* on a doubly logarithmic scale such that, for statistically self-similar curves, $L\left(k\right)\propto k^{-D}$. We used an open source Python implementation [53] and set $k_{max}=7$. The choice of $k_{max}=7$ was motivated by previous work showing a particularly accurate estimation of the fractal dimension in EEG signals with $k_{max}$ near 6 [23].

As HFD analysis is more effective and more efficient on shorter time windows [54] we use EEG data cropped to 2.0 s, non-overlapping, subsequent windows resulting in 50 × 2.0 s (100.0 s in total) repetitions from each of the EO and EC time series datasets recorded for all participants.

#### 2.4.3. Feature Extraction for Non-Oscillatory Features

A novel approach to feature extraction is proposed and implemented on non-oscillatory HFD features. The method normalises each participant’s EO EEG signal by using the amplitude of their corresponding EC EEG signal. The goal of this normalisation is to eliminate individual-specific differences in EEG amplitude, highlight differences between the EO and EC states while preserving phase information, and use a ratio of EO to EC data as a classifier input. Each signal, EO and EC, is a complex number and expressed as follows:

(6) $$X_{\mathrm{EC}}\left(f\right)=A_{\mathrm{EC}}\left(f\right)e^{j\phi_{\mathrm{EC}}\left(f\right)}$$

(7) $$X_{\mathrm{EO}}\left(f\right)=A_{\mathrm{EO}}\left(f\right)e^{j\phi_{\mathrm{EO}}\left(f\right)}$$

where $A_{\mathrm{EC}}\left(f\right)$ and $A_{\mathrm{EO}}\left(f\right)$ are the magnitudes, and $\phi_{\mathrm{EC}}\left(f\right)$ and $\phi_{\mathrm{EO}}\left(f\right)$ are the phases at frequency *f* for the EC and EO data, respectively. To normalise the EO signal by the EC signal, the amplitude of the EC signal is defined by:

(8) $$|X_{\mathrm{EC}}\left(f\right)|=A_{\mathrm{EC}}\left(f\right)$$

Three different normalisations were applied. The first, EO_*N*1_, is normalised by dividing EO by the amplitude of the EC signal given that EC power should always be larger than EO, meaning the resulting amplitude ranged from 0 to 1.

(9) $$\frac{X_{\mathrm{EO}}\left(f\right)}{|X_{\mathrm{EC}}\left(f\right)|}=\frac{A_{\mathrm{EO}}\left(f\right)}{A_{\mathrm{EC}}\left(f\right)}e^{j\phi_{\mathrm{EO}}\left(f\right)}$$

This may help to remove the influence of individual-specific amplitude characteristics by scaling the EO signal with respect to the EC signal. Importantly, the phase information of the EO signal, $\phi_{\mathrm{EO}}\left(f\right)$, is preserved. Additionally, to prevent division by zero when $A_{\mathrm{EC}}\left(f\right)$ approaches zero, a small constant ϵ is added to the denominator, where ϵ is a small value chosen to prevent instability in the calculation:

(10) $$\frac{X_{\mathrm{EO}}\left(f\right)}{|X_{\mathrm{EC}}\left(f\right)|+\epsilon }$$

For the second method, EO_*N*2_, the phase information of EO and EC is not retained. When transforming from the frequency domain to the time domain, the phase was set to 0 for all frequencies, meaning that only the amplitude information is preserved. Therefore, EO_*N*2_ is calculated as follows:

(11) $$\frac{|X_{\mathrm{EO}}\left(f\right)|}{|X_{\mathrm{EC}}\left(f\right)|}=\frac{A_{\mathrm{EO}}\left(f\right)}{A_{\mathrm{EC}}\left(f\right)}$$

The third method also set the phase to zero and calculates the ratio of the square values, EO_*N*3_:

(12) $$\frac{|X_{\mathrm{EO}}{\left(f\right)|}^{2}}{|X_{\mathrm{EC}}{\left(f\right)|}^{2}}=\frac{|A_{\mathrm{EO}}\left(f\right)\cdot e^{j\cdot \phi_{\mathrm{EO}}\left(f\right)}{|}^{2}}{|A_{\mathrm{EC}}\left(f\right)\cdot e^{j\cdot \phi_{\mathrm{EC}}\left(f\right)}{|}^{2}}=\frac{A_{\mathrm{EO}}{\left(f\right)}^{2}}{A_{\mathrm{EC}}{\left(f\right)}^{2}}$$

Normalised EO were then returned to the time domain and HFD values were extracted from 50 × 2 s repetitions from each set of data, the original EO, EO_*N*1_, EO_*N*2_, and EO_*N*3_.

### 2.5. Classification

#### 2.5.1. Classifiers

We explore the potential of band power and HFD as features for classification using two different algorithms: linear discriminant analysis (LDA) and linear support vector machines (SVM). Due to the small amount of data available, linear classifiers were selected given their relative simplicity compared to non-linear alternatives. Additionally, SVMs have been shown to outperform other methods in a large number of EEG classification problems, which is attributed, in part, to SVMs’ ability to classify relatively small datasets [55].

LDA attempts to find a linear combination of features that characterise or separate two different classes by projecting measurements onto another axis.

(13) $$h\left(t\right)=A^{t}\cdot f$$

where *f* is the original set of features, *A* is transformation, and *h*(*t*) is a linear discriminant function. The method aims to maximise the ratio of between-class variance to the within-class variance in any particular dataset in order to achieve maximal separability.

(14) $$\alpha =\frac{\sigma_{\mathrm{between}\;\mathrm{class}}^{2}}{\sigma_{\mathrm{within}\;\mathrm{class}}^{2}}$$

For the best separation of classes, alpha should be maximised. Sklearn’s implementation for LDA was used [56], setting prior probabilities to ’empirical’, meaning that the prior probability of class k is the number of training samples of class k, divided by the total number of training samples. LDA assumes that different classes have the same variance. Other parameters were kept constant with the following settings:

- Priors: Inferring prior probabilities from data—if not provided, the model estimates class probabilities directly from the training data distribution.
- Solver: SVD—singular value decomposition (SVD) is used for efficient computation, especially when the number of features is high.
- Shrinkage: None—no shrinkage is applied, meaning the model does not regularize the covariance matrix estimates.
- store_covariance: False—the covariance matrix is not stored in the model to reduce memory usage during training.

All LDA hyperparameters were fixed.

SVM classifier training involves representing the training data points in a multi-dimensional vector space and finding a hyperplane that separates the data points belonging to different classes, while maximising the distance between the hyperplane and the data points closest to it, which are referred to as ’support vectors’ in [57]. Here, we used the Sklearn implementation [58] of the ν-SVC classifier by Scholkopf et al. [57]. ν-SVC varied from 0.05 to 0.90 with a step size of 0.05. Other hyperparameters were kept constant with the following settings:

- Kernel: Linear—assumes data is linearly separable, reducing computational complexity.
- Probability: True—enables probabilistic outputs for predictions.
- tol: Default value $1\times 10^{-3}$—controls solver convergence by setting a minimum change threshold.
- max_iter: Default value −1—no iteration limit; solver runs until convergence is achieved.

#### 2.5.2. Classifier Evaluation

The following outcome measures were used to asses classification results [59]. For both algorithms outlined above, classification accuracy, sensitivity, and specificity were calculated as follows:

(15) $$Accuracy=\frac{\mathrm{TP}+\mathrm{TN}}{\mathrm{TP}+\mathrm{TN}+\mathrm{FP}+\mathrm{FN}}$$

(16) $$Sensitivity=\frac{\mathrm{TP}}{\mathrm{TP}+\mathrm{FN}}$$

(17) $$Specificity=\frac{\mathrm{TN}}{\mathrm{TN}+\mathrm{FP}}$$

where TP is true positive, TN is true negative, FP is false positive, and FN is false negative.

#### 2.5.3. Optimal Feature Selection

The input feature vectors for both classifiers were constructed from either feature (band power or HFD) extracted from a subset of EEG channels. Optimal EEG channels were selected via nested cross-validation on the training set, using a wrapper method for EEG channel selection known as *greedy forward feature selection* [60,61]. Greedy forward feature selection starts by estimating the cross-validation accuracy of the given machine learning algorithm (LDA or SVM) trained on extracted features from each EEG channel independently. The algorithm adds the channel with highest observed mean accuracy to the initially empty selected set. In each subsequent iteration, cross-validation accuracy of the classifier trained on each non-selected channel in conjunction with all the features in the selected set is evaluated, and the best performing further channel is added to the selected set. The procedure halts when there is no further channel to add without reducing classification accuracy. For a more detailed description and a pseudo-algorithm, see Chapter 7.3 in [60].

For SVM classification, the optimal ν-SVC hyper-parameter value ν∈(0,1], which bounds the fraction of support vectors and margin errors, was determined via a grid search and cross-validation of the training set, where the subset of channels used was re-optimised for each parameter value.

For the classification of HFD features extracted from EO, EO_*N*1_, EO_*N*2_, and EO_*N*3_ data, principal component analysis (PCA) [62] is applied during the greedy channel selection process to reduce dimensionality and use the resulting principal components as the classifier input. In this case, the input training data consist of a matrix structured as N_*patients*_ × N_*repetitions*_ × N_*channels*_ × *HFD*. PCA is applied to the channels and their corresponding HFD features and, as a result, the number of principal components is derived from this step and used as the input for the classifier.

Once PCA is applied to the training set, the same transformation (i.e., the derived PCA matrix) is applied to the test data. This ensures consistency, allowing the test data to be projected into the same feature space as the training data before being evaluated by the classifiers. This approach is applied to address potential overfitting to specific channels, and the greedy algorithm is designed to select the number of PCA components that provides the best cross-validation accuracy.

#### 2.5.4. Analysis Framework

**Figure 1:**
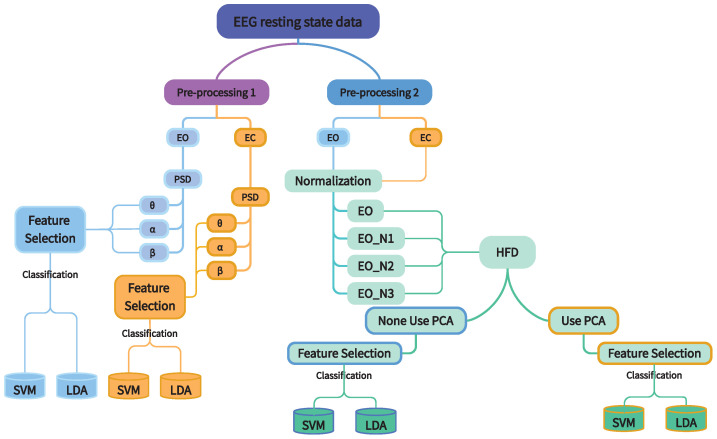
Schematic outlining all performed classification analyses in the study. Acronyms: EO, eyes opened; EC, eyes closed; $EO_{N1}$, $EO_{N2}$, $EO_{N3}$, different normalisation methods; SVM, support vector machine; LDA, linear discriminant analysis; PSD, power spectrum density; HFD, Higuchi fractal dimensions; PCA, principal component analysis.

Figure 1 outlines each EEG feature utilised and their subsequent classification. To explore whether extracted features can be used as susceptibility markers (i.e., markers of future pain) for CNP in different datasets, for each configuration show, LDA and SVM classifiers were first trained and tested on Dataset A to discriminate between PNP and PDP. Classifiers were trained on examples from all but one participant and evaluated on the held-out participant repeatedly, with each participant being held out once—also known as leave-one-out cross-validation (LOOCV)—on Datasets A or B separately. Next, to test the transferability of the designed methods and the usefulness of EEG features on an independent cohort of participants, where data were recorded under the same conditions, classifiers were then trained and evaluated using LOOCV of participants from Dataset A and B. Next, to understand whether optimal classifier parameters identified using one dataset (i.e., EEG channels, ν-SVC parameter value, the number of PCA components) could generalise to another, classifiers were trained using all available participants from Dataset A, validated using Dataset B, and vice versa.

## 3. Results

### 3.1. Classification Results

The classification with power band features is presented first, followed by the classification results with HFD.

#### 3.1.1. Band power Feature Classification

Table 1 outlines the classification performance of both the LDA and SVM models, evaluated across Dataset A and Dataset B under EO and EC conditions. For LDA, the highest accuracy was observed in Dataset A under EO conditions, achieving 80.0% ± 26.4% with a sensitivity of 79.0% ± 43.6% and specificity of 81.0% ± 44.7%, respectively. For SVM, the highest classification accuracy was observed in Dataset B under EO conditions, achieving 79.7 ± 25.8% with a sensitivity of 86.0 ± 45.50% and specificity of 73.0 ± 41.8%. Consistent performance was observed for both classifiers, although sensitivity and specificity varied more across datasets and conditions. When examining the channels selected during classification, no clear patterns emerged that would indicate any strong spatial preferences for either classifier across the different conditions. For both LDA and SVM, the selected channels are distributed across various regions of the scalp, covering the frontal, central, temporal, and occipital regions. Classification accuracy was better for EO than for EC for both datasets classified with LDA and for Dataset B classified with LDA.

The performance of the LDA and SVM classifiers assessed using combined Datasets A and B, focusing on the common set of 47 channels with LOOCV, can be seen in Table 2. These results are relatively lower than those observed in individual datasets, but performance is comparable between different classifiers and different features, with sensitivity and specificity having comparable values to accuracy. Frontal electrodes were most frequently selected. The best performance was achieved for SVM under EC conditions, reaching 72.4 ± 31.1% accuracy, 76.8 ± 43.5% sensitivity, and 68.0 ± 41.1% specificity. Three EEG electrodes were selected: one right frontal, one left parietal, and one right parietal. For LDA, marginally better classification was achieved under EO conditions, with 70.4 ± 31.4, 66.8 ± 39.8, and 74.0 ± 43.2% for accuracy, sensitivity, and specificity, respectively.

Table 3 presents the generalisability of classifiers trained on Dataset A by testing them using participant data from Dataset B, and vice versa. Validation metrics, and their respective st.dev, were calculated on a per-participant basis and averaged to obtain displayed performance. Classification accuracy was above chance (60.6 ± 33.4%) only for LDA classifier of EC features trained on Dataset A and tested on Dataset B. This is surprising given that dataset B was almost twice as large as Dataset A.

#### 3.1.2. HFD Feature Classification

The results obtained when classifying HFD features across individual datasets can be seen in Table 4. The LDA classifier achieved the highest accuracy of 72.7% ± 42.5% under EO conditions for Dataset A and 72.3% ± 22.8% under EC conditions for Dataset B. In contrast, SVM classifiers showed greater performance across both datasets, with accuracies reaching 85.0% ± 26.0% for Dataset A under EC conditions and 80.0% ± 31.0% for Dataset B under EO conditions. It is noteworthy that sensitivity and specificity were also higher on average for SVM than for LDA. Overall, using SVM for HFD feature classification outperformed LDA across the two datasets and conditions. Additionally, the SVM algorithm consistently selected one or two channels for classification where LDA classification required more channels to successfully distinguish between PDP and PNP. No clear localisation pattern emerges for the selected electrodes.

Table 5 shows the performance of classifiers assessed on Datasets A and B combined when using HFD features extracted from EO or EC states. Similarly to the results obtained on each dataset separately, the SVM classifier demonstrates significantly better performance, achieving 77.9% accuracy under EO conditions based on one EEG channel only, or 86.4% accuracy on the same dataset based on PCA derived from EEG electrodes. The LDA classifier achieved much lower accuracies of up to 66.2%.

#### 3.1.3. Normalisation and Ratio-Based Feature Classification (HFD)

SVM outperformed LDA in previous classifications; therefore, only SVM was considered in this section. Table 6 presents the performance of SVM classifiers trained using EO as well as normalised fetaures: EO_*N*1_, EO_*N*2_, and EO_*N*3_. Note that the whole Dataset A was tested, rather than individual participants; therefore, the standard deviation is missing. Sensitivity (Sen) and specificity (Spe) are also provided for each configuration.

The results show that all classifiers achieved a high leave-one-out testing accuracy between 76% and 100% for the training set, with as little as one EEG channel, with or without feature reduction using PCA. However, this high accuracy came at the cost of low generalisability, with most classifiers reaching chance levels.

The highest classification accuracy was achieved for EO_*N*3_ (square amplitude normalisation without phase) with PCA. This classifier used the largest number of EEG channels and related PCA that might reduce its overfitting to the training set. It is of interest to note that this classifier also achieved one of the highest leave-one-out testing accuracies on both Datasets A and B. Training on Dataset A and validation on Dataset B resulted in 66.6% validation and 100% test accuracy. Equally important from the overfitting perspective is the classifier for training Dataset A and testing on Dataset B, which achieved comparably high values for sensitivity (73%) and specificity (60.1%).

Validation of Dataset A, trained on Dataset B, also achieved an above-chance classification accuracy (59.5%), but sensitivity was low (37.5%). It is of interest that, for both band power and HFD features, better results were achieved for training and testing on Dataset A and validating on Dataset B, although Dataset B was larger. The reason might be that Dataset A has better separability of features. Supplementary Figure S3 shows the separability of band power features while Figure S4 shows the separability of HFD features in EO. For band power features, the uniform manifold approximation and projection method was used in order to combine multiple features, while HFD was presented in the form of a histogram. For Dataset A, HFD had slightly higher values (histogram shifted to the right), while for Dataset B, PDP had a binomial distribution with two peaks, one with lower and the other with higher HFD values than PNP. Lower separability between PNP and PDP in group B indicates less homogeneous grouping, which might be caused by recording in two different hospitals, i.e., in the presence of two different environmental noise levels, in contrast to Dataset A, which was all recorded in the same environment.

## 4. Discussion

In this study, we systematically analysed the generalisability of classifiers and EEG markers of future pain for people with subacute SCI. In our previous study, we achieved accuracies of 86 ± 10% (LDA) on the old Dataset A used in this study. By repeating the experiment with nearly double the number of participants and keeping experimental conditions as similar as possible, we created three levels of challenge:

- Classifying separately new and old datasets with the same type of EEG features and the same type of classifiers, but allowing the selection of optimal electrodes and network parameters for each set separately.
- Jointly classifying both datasets.
- Training and testing classifiers based on one dataset and validating on another.

We did not identify a single study in the recent published literature that tested the repeatability of chronic pain markers, i.e., all cross-sectional studies only addressed Challenge 1.

Most studies used simple classifiers due to smaller datasets, such as LDA, SVM, k nearest neighbours, naïve Bayesian, and random forest, though perceptron NNs [7] and convolutional neural networks [32] were also applied. EEG has been used to identify different types of chronic pain, including hip pain with an accuracy of 65% [35], chronic pancreatitis with an accuracy of 87.5% [36], lower back pain with accuracies up to 83% [15,32], and peripheral neuropathy with an accuracy of 80% [37]. Studies aimed at classifying mixed types of chronic pain based on EEG achieved variable accuracies ranging from 57% [41] to 92.5% [42]. Most frequent features were based on band power, but entropy, connectivity, and derived PCA were also used.

The main difference between the previous studies and the current study is that none of the groups in this study actually had pain at the time of EEG recording. Still, our study achieved comparable accuracies to those of other studies, even when new and old datasets were combined.

To create susceptibility markers, three aspects had to be considered: features (PSD and HFD), classifiers (LDA, SVM), and resting state conditions (EO and EC). We will first comment on general results common to all three levels of challenge.

Two sets of features were derived in two different domains: linear features based on band power were calculated in the frequency domain and non-linear HFD features were calculated in the time domain. The other relevant difference between linear and non-linear features was that the former were calculated based on 10 s segments to improve the signal-to-noise ratio, while the latter were calculated on 2 s segments, as recommended in the literature [63]. That resulted in effectively 5 times more HFD features, albeit potentially at the cost of a lower signal-to-noise ratio. This could partially explain the better results obtained with HFD features.

We looked at the feature distribution (HFD) of both datasets that we presented in the Supplementary Materials. Band power features seem to be better separated in Dataset A than in Dataset B. In addition, for HFD of Dataset B, the PDP group had a bimodal distribution. This might explain the slightly lower classification accuracy and the fact that training on Dataset B resulted in poor validation on Dataset A.

Recently, Zollezi et al. [64] compared linear (band power) and non-linear (entropy) EEG features, both calculated over different frequency bands. Although they did not perform classification, statistical analysis showed that non-linear features were able to better predict different levels (low, medium, and high) of pain severity. In the current study, better classification was achieved with non-linear features, even when PCA was not derived.

In the literature, most studies use simple classifiers due to smaller datasets, such as LDA, SVM, k nearest neighbours, naïve Bayesian, and random forest [65]. Classifiers selected in this study were relatively simple and recommended for the classification of smaller datasets. While SVM is more widely used for EEG classification of pain [6,17,45,48], its disadvantage is that it contains hyperparameter ν, which critically affects the performance of the classifier. In this study, and in particular for HFD features, SVM outperformed the LDA. After normalisation, accuracy higher than 90% was achieved, indicating that for this problem, simple classifiers have a comparable performance to that of classifiers based on NN. We achieved classification accuracies between PNP and PDP in Dataset A of up to 89% using the perceptron network, although classification accuracies of up to 86% were achieved with LDA [7]. In other studies, NNs such as convolutional NNs [32] (accuracy 83%), elastic net [43] (accuracy 79.6%), or STPA net [44] (accuracy 87.8%) were used to detect different types of chronic pain. Results from the literature show that simpler and NN-based classifiers achieve comparable accuracies. However, more complex NN classifiers might be more prone to overfitting due to small sizes of datasets. For larger datasets, deep NN-based classifiers would have multiple advantages such as sensor or feature fusion [66], integration of feature selection with classification [67], or feature agnostic classification [68].

When addressing the first challenge, all combinations of features, classifiers, and resting state conditions resulted in comparable high performances on both datasets, showing that future NP pain indeed creates changes in EEG that could be identified in both the time and frequency domains. The lack of any preferable spatial location indicates the widespread nature of these changes.

When addressing the second challenge, classification accuracy dropped only slightly, reaching a maximum of 72% for band power and 77.9% for HFD features. These results show that, despite the stochastic nature of EEG, the features that characterise future pain are robust and could also be identified in both time and frequency. Non-linear features outperformed linear features, and SVM clearly outperformed LDA for non-linear features, because it allowed an additional level of flexibility compared to LDA. The EO state, as hypothesised, provided more informative features than EC, resulting in a 12% higher classification accuracy. While a small number of selected channels indicates potentially simpler setups for future clinical applications, it might also increase the chances of overfitting. The third challenge was designed to test for this. Unfortunately, when using EO or EC features separately, only band power with LDA reached an accuracy above the chance level. This indicates the tendency of overfitting, which had to be addressed by introducing variability and also increasing separability between different classes. A novel normalisation procedure introduced in this study served several purposes. Knowing that EC EEG amplitude is typically larger than EO amplitude, by dividing EO with EC we, reduced the range of EEG signal amplitudes, which may vary considerably between individuals. This might not be the most critical step, considering that HFD are derived features, although there is a link between FFT amplitude and frequency and HFD [63]. Secondly, because there is no direct 1:1 link between EO and EC segments, this allows the creation of multiple surrogate data, a feature that should be exploited more in future studies. Thirdly, and possibly most relevant for these datasets, by introducing the EO/EC ratio rather than considering each state separately, it is likely that we increased the separability of features between PDP and PNP. This is because the reactivity to eyes opening (and consequently the EO/EC ratio) is reduced in the presence of current or future pain.

Three different normalisation procedures were introduced. It is of interest that normalisation with square amplitude and removing the phase information superseded the testing accuracy of the normalisation with the preserved phase information. Subsequent feature reduction with PCA improved the validation accuracy. While classification was well above the chance level (66.6%), there is clearly room for improvement to make it suitable for practical use.

In the future, normalisation could also be applied to frequency domain features. In our previous study [7], we used the EEG EO/EC power ratio as one of the features alongside EEG power in different frequency bands. Varying the duration of EEG sequences or creating overlapping segments might, in the case of PSD, increase the number of training sets. In that case, more complex classifiers based on NN could be applied. Feature fusion (oscillatory and non-oscillatory) could be another avenue to improve the generalisability of classification. Alternatively, combining EO and EC features might also lead to better accuracy. Ultimately, the best way to improve classification performance would be to collect a larger dataset and to use an NN, which would enable combined classification and feature selection and also allow for testing whether creating synthetic EEG from the existing Datasets A and B would lead to a larger dataset suitable for NN classifiers. The main purpose of an NN would be to improve validation accuracy, considering that SVM already provided very high training and testing accuracy. From a data collection perspective, ideally, each participant should be reassessed by the experimenter in person to establish whether they developed NP, and an EEG should be recorded again on that occasion. In that way, it would be possible to determine the EEG features that changed the most due to NP. Currently, the best approximation of this would be to compare EEGs in PDP with those of patients who already had pain at the time of recording, as demonstrated for Dataset A [7]. This comparison showed that these two groups could be separated.

A limitation of the study is the size of the datasets, although together they are among the larger datasets in the literature (N = 52). A larger dataset would be more suitable for NN classifiers, which might result in better validation performance. Another limitation is that we determined pain based on patient records combined with phone calls rather than on in-person assessments by experimenters, which might provide better patient profiling but would be logistically challenging, given the large catchment areas of the hospitals (e.g., one hospital for the whole Scotland). Participants were initially recruited while they were still hospitalised. A realistic challenge that might have affected the separability of Dataset B features was the different environmental noise levels present at the two hospitals from which the patients were recruited (one in England and the other in Scotland), although the same portable EEG was used to avoid issues that might arise due to different performances of EEG datasets from two manufacturers or slightly different amplifications of two EEG devices from the same manufacturer. Finally, although we followed the same procedures for collecting Dataset B and Dataset A, different experimenters collected them; therefore, some variation in the experimental protocol was inevitable.

As mentioned above, we recorded EEGs in four different groups and selected two of the most challenging groups. In the future, generalisability should be tested between other groups, like patients who had pain and able-bodied people without pain or patients who had pain during EEG recording and those who later developed pain.

## 5. Conclusions

Repeated experiments confirmed that susceptibility markers of future pain exist and that they can be identified based on both linear and non-linear EEG features. Classification of the combined Datasets A and B further confirmed the robustness of future pain features that could be identified despite the presence of different environmental noise in the two datasets. Utilising inherent differences between the EO and EC states between the no pain and future pain groups ultimately improved the generalisability of the classifier based on time domain features. Normalising EO with EC features opened the pathway to creating surrogate data and applying more complex classifiers. Future research should focus on improving validation performance as a prerequisite for real life applications.

## Figures and Tables

**Table 1:** Classification performance of Linear Discriminant Analysis (LDA) and Support Vector Machine (SVM) models on individual datasets under both EO and EC conditions using theta, alpha and beta band power features. The results are presented as mean accuracy, sensitivity, and specificity ± st.dev (%), along with the optimal channels selected for each classifier and the ν parameter value where applicable.

	Dataset	State	Accuracy (%)	Sensitivity (%)	Specificity (%)	Channels	ν
LDA	A	EO	80.0 ± 26.4	79.0 ± 43.6	81.0 ± 44.7	Fpz, F8, T7, T8, C5, C6, CP1	-
		EC	70.0 ± 28.9	60.0 ± 37.7	78.0 ± 41.5	FC5, C6, Cz, CP3	-
	B	EO	76.7 ± 29.1	76.0 ± 43.0	77.3 ± 44.0	CP1, CP2, CP4, CP5	-
		EC	72.8 ± 28.2	72.9 ± 43.7	72.7 ± 39.0	Oz, F3, C5, T8, T7, FC5, CP1	-
SVM	A	EO	75.5 ± 28.9	87.0 ± 45.1	64.0 ± 39.8	FC3, P6	0.15
		EC	78.9 ± 20.5	75.0 ± 39.0	82.0 ± 43.9	Fp1, C1, P6	0.05
	B	EO	79.7 ± 25.8	86.0 ± 45.5	73.3 ± 41.8	CP6, T7, FC6, F8, O1, F1, F7, CPz, C4	0.05
		EC	70.9 ± 28.9	74.7 ± 41.5	66.7 ± 39.9	FC2, T8, FC3, C5, P6, C2	0.50

**Table 2:** Classification performance of both linear discriminant analysis (LDA) and support vector machine (SVM) models using combined Datasets A and B with a common set of 47 channels and theta, alpha, and beta band power features extracted from both EO and EC states. The results are presented as mean accuracy, sensitivity, and specificity ± st.dev (%), along with the optimal channels selected for each classifier and the ν parameter value for SVM classifiers, where applicable.

	Dataset	State	Accuracy (%)	Sensitivity (%)	Specificity (%)	Channels	ν
LDA	A & B	EO	70.4 ± 31.4	66.8 ± 39.8	74.0 ± 43.2	F2, F4, F7, FC3, T7, CP5, P8	-
		EC	69.2 ± 30.6	68.4 ± 40.9	70.0 ± 40.8	FC3, C6, P6, P8	-
SVM	A & B	EO	69.6 ± 33.4	80.0 ± 44.8	59.2 ± 38.5	C4, P4	0.45
		EC	72.4 ± 31.1	76.8 ± 43.5	68.0 ± 41.1	F2, P5, P6	0.15

**Table 3:** Classification performance of linear discriminant analysis (LDA) and support vector machine (SVM) models trained on either Dataset A or B and tested on the other validation dataset. Optimal channels and ν values (for SVM) were identified using the training dataset. Theta, alpha, and beta band power features were extracted from both EO and EC conditions. The results are presented as mean accuracy, sensitivity, and specificity ± st.dev (%).

	Train Dataset	State	Accuracy (%)	Sensitivity (%)	Specificity (%)
LDA	A	EO	46.7 ± 36.3	39.3 ± 30.8	54.0 ± 37.8
		EC	60.6 ± 33.4	55.3 ± 38.2	52.0 ± 36.9
	B	EO	49.5 ± 38.1	65.0 ± 44.0	34.0 ± 24.9
		EC	43.9 ± 36.5	31.3 ± 24.3	54.0 ± 39.7
SVM	A	EO	49.3 ± 42.3	72.7 ± 44.1	26.0 ± 28.2
		EC	52.2 ± 40.9	24.7 ± 27.0	83.3 ± 44.4
	B	EO	52.0 ± 33.7	62.0 ± 38.1	42.0 ± 31.4
		EC	46.7 ± 40.0	67.5 ± 42.6	30.0 ± 28.1

**Table 4:** Classification performance of linear discriminant analysis (LDA) and support vector machine (SVM) models on individual datasets under both EO and EC conditions using features based on Higuchi fractal dimension (HFD) analysis. The results are presented as mean accuracy, sensitivity, and specificity ± st.dev (%), along with the optimal channels selected for each classifier and the ν parameter value, where applicable.

	State	Train Dataset	Accuracy (%)	Sensitivity (%)	Specificity (%)	Channels	ν
LDA	EO	A	72.7 ± 42.5	67.0 ± 38.0	77.0 ± 42.0	Fp1, F7, F2, F8, FC3, T8, CPz, P3, Oz	-
		B	67.0 ± 27.6	68.0 ± 38.0	66.0 ± 40.0	F8, FC6, C5, T8, P2, P4, P8, O2	-
	EC	A	60.9 ± 35.0	44.0 ± 33.0	74.0 ± 42.0	CP4	-
		B	72.3 ± 22.8	75.0 ± 39.0	70.0 ± 40.0	C6, P6, PO4	
SVM	EO	A	88.6 ± 23.3	94.0 ± 47.9	84.3 ± 46.5	CP4	0.15
		B	76.3 ± 29.6	70.7 ± 43.3	81.8 ± 43.6	CP3, CP4	0.40
	EC	A	80.2 ± 33.1	67.3 ± 43.3	90.5 ± 47.2	F4	0.20
		B	77.4 ± 39.7	68.2 ± 45.6	86.7 ± 49.6	FC5	0.15

**Table 5:** Classification performance of both linear discriminant analysis (LDA) and support vector machine (SVM) models using combined Datasets A and B with a common set of 47 channels and Higuchi fractal dimension (HFD) features extracted from both EO and EC states. The results are presented as mean accuracy, sensitivity, and specificity ± st.dev (%), along with the optimal channels selected for each classifier and the ν parameter value, where applicable.

	State	Train Dataset	Accuracy (%)	Sensitivity (%)	Specificity (%)	Channels	ν
LDA	EO	A & B	60.7 ± 26.4	56.0 ± 34.4	65.0 ± 36.4	Fp1, CP1, P1, P2, PO4, O2	-
	EC	A & B	66.2 ± 33.0	64.0 ± 39.0	68.0 ± 41.0	F2, F4, FC2, T7, C3, T8, P4, FC5	-
SVM	EO	A & B	77.9 ± 37.8	87.8 ± 48.2	68.7 ± 46.0	P2	0.85
	EC	A & B	77.0 ± 35.4	70.7 ± 42.1	82.9 ± 49.1	Pz	0.80

**Table 6:** Performance of the support vector machine (SVM) classifier trained on either Dataset A or B and tested using participants in the alternate dataset. The results are shown for the Higuchi fractal dimension (HFD) extracted from the EO feature set and normalised EO_*N*1_, EO_*N*2_, and EO_*N*3_ features. Training and validation accuracies are provided for each configuration, and with and without use of principal component analysis (PCA). Train-acc, training accuracy obtained using leave-one-out cross-validation (LOOCV) on specified dataset; Val-acc, mean accuracy obtained when testing on participant data within the validation dataset. Channel selections, the number of PCA components, and the ν parameter value informed during training using the specified training dataset. All results are presented as mean ± st.dev.

	Dataset	PCA Comps	Train-acc (%)	Val-acc (%)	Sen (%)	Spe (%)	Channels	ν
EO	A	None	88.6 ± 23.3	48.3	51.1	45.5	CP4	0.15
	B		76.3 ± 29.6	43.7	98.0	0.02	CP3, CP4	0.40
	A	1	89.1 ± 21.4	45.2	73.5	16.8	Fp1, FC5	0.75
	B	1	80.8 ± 32.2	52.8	34.7	67.3	FC4, PO3	0.25
${\mathrm{EO}}_{N1}$	A	None	81.9 ± 36.8	49.4	71.8	30.0	T7	0.05
	B		89.4 ± 30.0	45.3	98.6	2.7	PO4	0.80
	A	1	91.9 ± 18.6	50.0	0.0	100.0	C1, O1	0.70
	B	1	83.3 ± 32.8	54.3	17.0	84.1	Fp2, P4	0.80
${\mathrm{EO}}_{N2}$	A	None	87.9 ± 31.3	47.7	93.1	2.1	Fp1, P2	0.25
	B		84.0 ± 30.0	44.4	100.0	0.0	CP6	0.20
	A	1	93.4 ± 23.0	50.9	87.9	13.9	F7, F4, P4	0.70
	B	1	90.3 ± 27.4	44.4	100.0	0.0	FC2, C1, O1	0.85
${\mathrm{EO}}_{N3}$	A	None	94.3 ± 22.9	50.0	100.0	0.0	CP4	0.15
	B		83.3 ± 37.2	45.8	37.5	52.5	F4	0.05
	A	8	100.0 ± 0.0	66.6	73.0	60.1	F3, FC2, T7, C2, CP4, Pz, P2, O2	0.20
	B	9	93.3 ± 24.9	59.5	37.5	77.0	Fp1, F3, Fz, FC1, C4, T8, CP4, P7, P5	0.45

## Data Availability

Dataset A is available at https://enigma.ini.usc.edu/ongoing/enigma-chronic-pain/, (accessed on 21 November 2024). Dataset B is available on request.

## Supplementary Materials

The following supporting information can be downloaded at: https://www.mdpi.com/article/10.3390/biomedicines13010213/s1, Table S1: Demographic information of SCI patients from all groups; Figure S1: (a) Electrode locations for dataset A, (b) Electrode locations for dataset B; Figure S2: Flow chart showing all patient groups in datasets A and B and patients groups analysed in this study; Figure S3: UMAP Visualisations of EO/EC alpha bandpower features extracted from PDP and PNP groups from (a) Dataset A. (b) Dataset; Figure S4: HDF histograms with PnP and PdP data: (a) group A of EO data. (b) group B of EO data.
