# Supplementary material for: Generalisation of EEG-Based Pain Biomarker Classification for Predicting Central Neuropathic Pain in Subacute Spinal Cord Injury
Source: Biomedicines. 2025 Jan 16;13(1):213. doi: 10.3390/biomedicines13010213 (PMC11759196; doi:10.3390/biomedicines13010213)
Supplement: Supplementary file 1 [file biomedicines-13-00213-s001.zip › biomedicines-3374186-supplementary.pdf]

Table S1: Demographic information of SCI patients from all groups. ASIA Classification: A: complete sensory and motor loss; B: incomplete sensory, complete motor loss; C and D: incomplete sensory and motor loss. Level of injury: C cervical, T thoracic, L lumbar.

Group	Subgroup	No.	Age	Weeks after injury	ASIA Classification	Level of Injury
PNP	Group A	1	51	12	D	T7, T10
		2	22	12	B	L1
		3	47	7	D	T11
		4	41	4	A	T12
		5	59	12	A	T6
		6	43	21	B	T6/T7
		7	24	7	A	L1
		8	38	4	A	L1
		9	62	10	A	T3, T5
		10	34	10	A	T6
	Group B	1	64	7	C	T11/T12
		2	51	5	C	C2
		3	23	11	C	L1-L3
		4	63	17	C	C3/C4
		5	51	11	D	T4-T9
		6	67	13	C	C4/C5
		7	49	16	C	C6/C7
		8	66	22	C	C2-C6
		9	52	10	A	T9
		10	44	7	B	C5
		11	58	18	D	T3
		12	54	16	B	T8
		13	75	5	D	C4/5
		14	72	20	D	C4/5
		15	55	16	D	C7, T3/4
PDP	Group A	1	70	9	D	T7/T8
		2	49	6	A	T12
		3	19	12	A	C5/C6
		4	69	6	B	L2
		5	32	24	A	T3
		6	46	6	A	T5
		7	49	4	A	T6
		8	32	6	A	C3
	Group B	1	62	8	D	C5/C6
		2	39	13	A	T3-T5
		3	56	23	A	T4/T5
		4	65	18	C	C3
		5	68	14	D	C2
		6	75	21	C	C3-6
		7	74	20	D	C6/7
		8	72	14	D	C3/4
		9	74	16	D	C5
		10	24	20	C	C3
		11	75	26	D	C3
		12	61	18	A	T6
		13	41	19	D	C3-5
		14	21	18	B	T6
		15	56	30	-	-
		16	62	20	B	C4
		17	58	19	D	T4

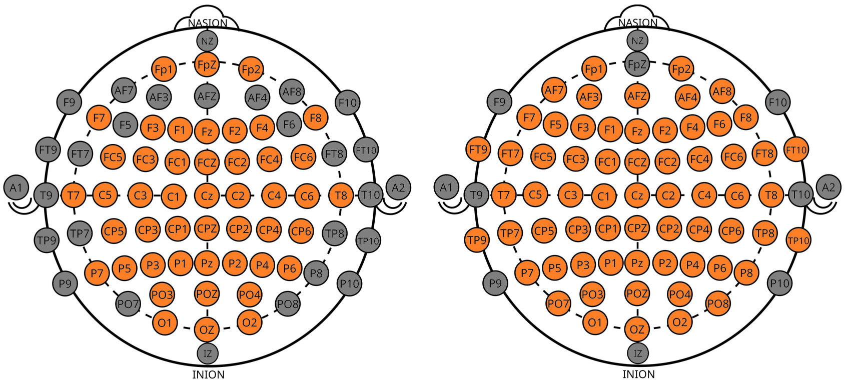

((a)) ((b))

Figure S1: (a) Electrode locations for dataset A, (b) Electrode locations for dataset B

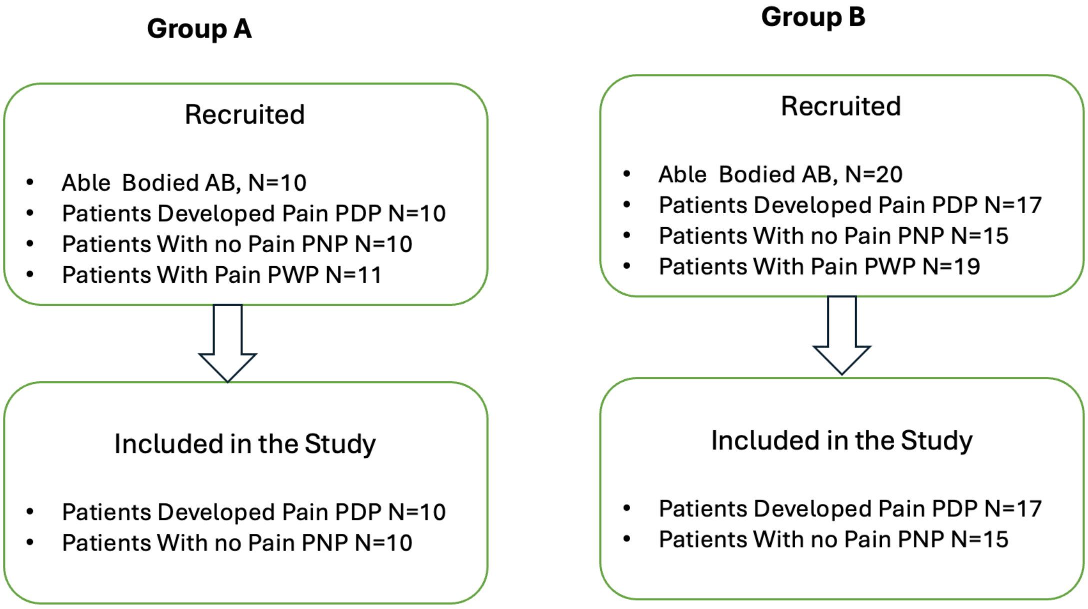

Figure S2: Flow chart showing all patient groups in datasets A and B and patients groups analysed in this study.

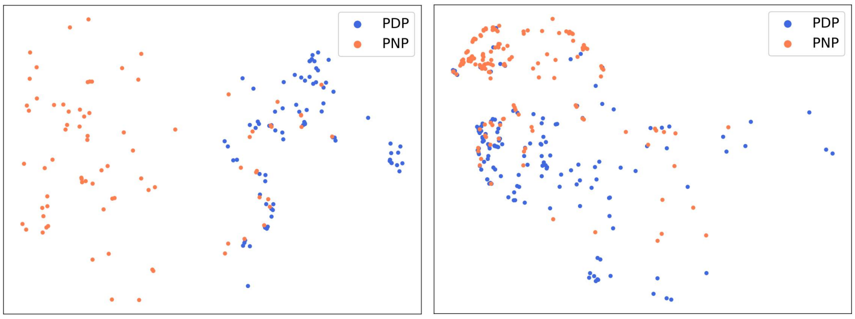

((a)) ((b))

Figure S3: UMAP Visualisations of EO/EC alpha bandpower features extracted from PDP and PNP groups from (a) Dataset A. (b) Dataset.

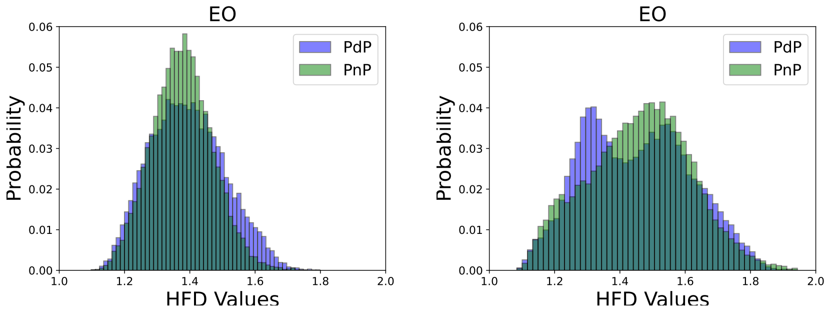

((a)) ((b))

Figure S4: HDF histograms with PnP and PdP data: (a) group A of EO data. (b) group B of EO data.
